# Gail Borden’s Great Inventions

**Published:** 1876-12

**Authors:** 


### *GAIL BORDEN'S GREAT INVENTIONS.*

They are wonderfully convenient these articles from the Borden Company. We use their condensed milk daily, and should hardly know how to keep house without it. When we were living in a tent at Ocean Grove last summer we found the condensed milk, condensed coffee, condensed cocoa and extract of beef, all that were needed in their respective lines. All these come in cans, are pure and genuine, will keep anywhere, and, though luxuries, are not costly.

